# Fecal Genetic Mutations and Human DNA in Colorectal Cancer and Polyps Patients

**DOI:** 10.31557/APJCP.2019.20.10.2929

**Published:** 2019

**Authors:** Jacqueline Lima, Yolanda Teixeira, Célia Pimenta, Aledson Vitor Felipe, Tiago Donizetti Silva, Ermelindo Della Libera Junior, Sarhan Sydney Saad, Elisabeth Deak, Helena Murray, Nora Manoukian Forones

**Affiliations:** 1 *Oncology Group, Gastroenterology Division, Medicine Department, *; 2 *Gastroenterology Division and Endoscopist,*; 3 *Surgery Department, Universidade Federal de Sao Paulo, São Paulo, Brazil, *; 4 *Randox Laboratories Ltd., Crumlin, Co. Antrim, United Kingdom.*

**Keywords:** Stool DNA, colorectal cancer, polyps, colonoscopy, screening

## Abstract

**Background:**

Colorectal cancer (CRC) is one of the most frequent cancers. Genetic mutations in CRC already described can be detected in feces. Microarray methods in feces can represent a new diagnostic tool for CRC and significant improvement at public health. **Aim: **to analyze stool DNA by human DNA quantify and microarray methods as alternatives to CRC screening. Method: Three methods were analyzed in stool samples: Human DNA Quantify, RanplexCRC and *KRAS/BRAF/PIK3CA (KBP) *Arrays.

**Results:**

KBP array mutations were presented in 60.7% of CRC patients and RanplexCRC Array mutations in 61.1% of CRC patients. Sensitivity and specificity for human DNA quantification was 66% and 82% respectively. Fecal KBP Array had 35% sensitivity and 96% specificity and RanplexCRC Array method had 78% sensitivity and 100% specificity.

**Conclusion:**

Microarray methods showed promise as potential biomarkers for CRC screening; however, these methods had to be optimized to improve accuracy and applicability by clinical routine.

## Introduction

Colorectal cancer (CRC) is one of the most frequent cancers worldwide. It is the second most common cancer in Brazil, with estimated risk of 36360 new cases (INCA, 2016). The most effective way to treat CRC is surgery resection, but many patients die from disease progression. Risk of developing CRC increases with age, inadequate living habits and inherited genetic mutations are other factors that can increase risk of CRC (NCCN, 2018). 

Early CRC detection can be accomplished through screening programs that reduce incidence and mortality rates (Lee et al., 2014, Schreuders et al., 2015). Fecal Occult Blood Testing (FOBT) by immunological test (FIT) or guaiac method (Lee et al., 2014) and colonoscopy are the most common screening test for CRC and polyps. Colonoscopy reduce incidence by 40% through the polyps removal but is an invasive and expensive method (NCCN, 2018). 

Concomitantly, several studies aiming to find other tests capable of detecting mutations in colorectal carcinogenesis are being developed to improve the chance to trace the cancer (Itzkowitz et al., 2007, AHRQ, 2012, MLDT, 2014). Studying altered human DNA in stool for CRC identification has been described since 2000 (Ahlquist et al., 2000). 

Approximately 0.01% of DNA found in stool is of human origin. Potential biomarkers for screening of CRC such as *KRAS*, *TP53* and *BAT26*, among other genes, are being analyzed and considered as stool markers complementary to existing methods of cancer identification (Vaughn et al., 2011, Tian et al., 2013, Colucci, 2013). Currently, the commercially available in the United States called Cologuard® test analyzes *KRAS* mutations, NDRG4 and BMP3 methylation and *ACTB* gene (MLDT, 2014).

The study of DNA by fecal samples is a non-invasive method and can have higher adhesion and indication even in ageing patients or with serious comorbidities (MLDT, 2014, Itzkowitz, 2007).

The purpose of this study was to analyze stool DNA by human quantification and mutation microarray methods as alternatives to colorectal cancer screening.

## Materials and Methods

The study had been approved by a local committee No. 1,173/09. All subjects were informed of study objectives, collection procedures and signed a consent form. 

Patients collected stool samples at home, prior to colonoscopy, froze them (-20°C) until delivery to Molecular Gastro-oncology Laboratory of Universidade Federal de São Paulo. During colonoscopy biopsies from cancer, polyps and normal tissue were collected. In cancer patients, tumor tissue was also collected during surgery. Patients with inflammatory bowel disease or other cancers were excluded.

DNA from tissue and fecal samples were extracted, quantified and genotyped by the Evidence Investigator^TM ^*KRAS*/*BRAF*/*PIK3CA* Array (KBP Array) (Randox Laboratories Ltd, Crumlin, Northern Ireland) method. In some tissue, mutations were confirmed by Sanger sequencing. Fecal DNA mutations were also surveyed by Evidence Investigator^TM^ RanplexCRC Array (Randox Laboratories Ltd, Crumlin, Northern Ireland) method. Human DNA in stool was measured by Quantifiler™ Human DNA Quantification Kit (ThermoFisher Scientific, Waltham, Massachusetts, EUA).

A total of 152 individuals were included: 60 of them collected stool samples, 42 collected tissue samples and 50 collected both samples, totaling 110 stool samples and 92 tissue samples. 

Human DNA quantification was performed at Molecular Gastro-oncology Laboratory, UNIFESP. Array tests were performed within the Molecular Biology R and D Department of Randox Laboratories Ltd, Crumlin, Northern Ireland. 

Tissue samples were stored at -80°C. DNA extraction followed manufacturer’s protocol for DNA isolation obtained from human tissues (QIAGEN 56304 - QIAamp DNA Micro Kit, Hilden, Germany). 

The stool samples delivered were stored at -80ºC. DNA extraction was performed following manufacturer’s protocol for DNA isolation obtained from human feces for analysis (QIAGEN 51504 - QIAamp DNA Stool Mini Kit, Hilden, Germany). DNA was extracted in triplicate for each stool sample, as recommended by the RanplexCRC Array protocol and stored at -20°C until subsequent mutational analysis. Total DNA present in feces and DNA extracted from colonic tissues were quantified by spectrophotometry at 260 nm (NanoDrop 1000 - Thermo Fisher Scientific, Waltham, Massachusetts, EUA).

DNA extracted from fecal samples were also mixed in the same tube to form a pool of stool DNA from everyone. A second measurement of pool samples were performed by spectrophotometry at 260 nm.

Human DNA was quantified in DNA pool for each patient by Real-Time PCR (Real Time PCR System StepOnePlus, Applied Biosystems, Foster City, Califórnia, EUA). One Standard Human Quantifier (Applied Biosystems, Foster City, Califórnia, EUA) human quantification kit was used.

Mutations in TP53, *KRAS*, *BRAF* and APC genes in stool DNA were studied following manufacturer’s protocol of Evidence Investigator^TM^ RanplexCRC Array based on 28 simultaneous mutation detections in these genes by microarray. The protocol is based in DNA extraction, probe hybridisation, ligation, PCR amplification and microarray hybridisation, optimized for the qualitative assessment stool DNA.

The Evidence Investigator^TM^ KBP Array detects 20 mutations in *KRAS*, *BRAF* and *PIK3CA* genes in DNA extracted from CRC tissue. The manufacturer’s protocol was followed accordingly. Note that this method has been optimized for use with CRC tissue (fresh/frozen tissue or FFPE) and not for stool or polyps. The possibility to use this method with stool DNA samples may contribute to screening for colorectal cancer. Sanger sequencing was used to confirm positive mutations in DNA from target tissue analyzed by the KBP Array.

*Statistical Analysis*

Parametric statistical tests were used because data is quantitative and continuous. ANOVA and T-test for means comparison based on variance. Concordance Index was performed to measure degree of agreement between two variables and/or results. In interpreting results, Kappa <20% was considered negligible; 21 to 40% minimum; 41 to 60% regular; 61 to 80% good; above 81%, great. In comparison between two or more variables and/or their levels, chi-square was performed. ROC curve was performed to determine cut off level for human DNA in stool samples with greatest sensitivity and specificity. A significance level of 5% (p=0.05) was defined for this assessment and confidence intervals constructed throughout the work were set at 95%.

## Results

Clinical and epidemiological characteristics from the cohort of 152 patients were divided into 3 groups: 60 individuals with normal colonoscopy (controls), 32 subjects with polyps (75.8% adenoma polyps and 24.2% hyperplastic polyps, 64% had ≤1 cm) and 60 with CRC. The mean age was 60.2, 68.1, 63.4, respectively.

**Table 1:** Mutations by KBP Array in Tissue and in the Stools among the Groups

KBP Array	Control	Polyp	CRC		CRC vs Control	CRC vs Polyp	Control vs Polyp
				p-value	p-value	p-value	p-value
Tissue							
WT (n=62)	34 (54.8)	15 (24.2)	13 (21.0)	< 0.001	< 0.001	0.593	< 0.001
MUT (n=30)	2 (7.0)	12 (40.0)	16 (53)				
* KRAS*	-	10 (29.4)	14 (41.2)	0.116			
* BRAF*	2 (5.9)	4 (11.8)	2 (5.9)				
* PIK3CA*	-	1 (2.9)	1 (2.9)				
≥ 2 MUT	-	3 (8.8)	1 (2.9)				
Stool							
WT n= 62	26 (41.9)	4 (6.5)	32 (51.6)	<0.001	0.006	0.032	<0.001
MUT n= 28	1 (3.6)	10 (35.7)	17 (60.7)				
* KRAS*	-	1 (3)	17 (51.6)				
* BRAF*	1 (3)	10 (30.3)	-				
* PIK3CA*	-	-	4 (12.1)				
≥ 2 MUT	-	1 (3)	4 (12.1)				

CRC, colorectal cancer; MUT, mutated; WT, Wild-type; *Inconclusive tests were excluded

**Figure 1:**
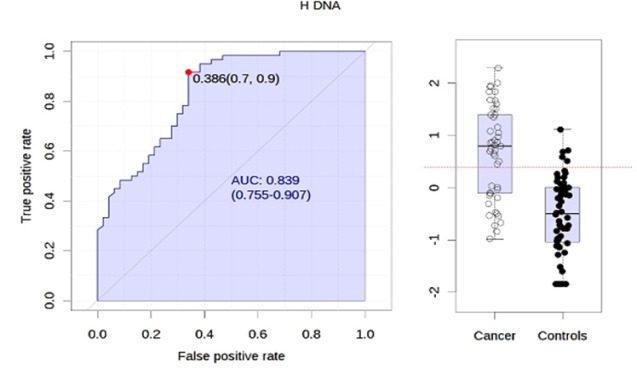
Cutoff Value of Human DNA Quantification by ROC Curve

**Table 2:** Comparison KBP and RanplexCRC Methods in Stool Samples

	Ranplex CRC n (%)	KBP n (%)	p-value*	KAPPA %	p-value**
Total					
WT	10 (20.8)	28 (58.3)	<0.001	44.5	0.001
MUT	18 (37.5)	15 (31.3)			
INC	20 (41.7)	5 (10.4)			
CRC					
WT	10 (20.8)	14 (29.2)	0.368	64	<0.001
MUT	7 (14.6)	5 (10.5)			
INC	3 (6.2)	1 (2.1)			

INC, Inconclusive; WT, wild-type, * Qui square test, ** Kappa test

**Table 3:** Sensitivity, Specificity, Positive and Negative Predictive Value for Cancer Diagnosis KBP Methods and RanplexCRC*

	Sensitivity %	Specificity %	PPV %	NPV %

Human DNA Quantify	66	82	74	75
KBP Tissue	55	94	89	72
KBP Fecal	35	96	94	45
KBP fecal (DNA>0.4 ng/ml)	56	69	72	53
Ranplex CRC	78	100	100	57

NPV, negative predictive value; PPV, positive predictive value; * Excluded inconclusive results.

Among the stool samples, 47 were from CRC patients, 44 controls and 16 were from patients with polyps. The total DNA found in feces was not different between the groups. In regards to human DNA quantification (HDNA), we observed an average 0.46 ng/µl for controls, 15.05 ng/µl for CRC and 0.10 ng/µl for polyps (p <0.0001). The percentual of HDNA percentage in the CRC group, control group and polyp group were 0.4%, 0.12% and 16.5%, respectively of total DNA. The mean HDNA was higher in tumors localized in the left and rectum (18.54±33.24 vs 0.31±0.57, p=0.002). There was no difference between the stages (p=0.247).

*KBP Array in Tissue and Stool DNA*

From the 92 tissue samples analyzed, 30 had one or more mutations and 62 had wild-type (WT). The mutations occurred in 53% in CRC, 40% in the polyp group and 7% in controls. *KRAS* gene mutations were the most prevalent, mainly in the CRC group (41.2%) (Table 1). 

Genetic sequencing was performed in 29 (97%) tissue samples with mutation by KBP Array. Twenty-two samples had mutation confirmed by Sanger. *KRAS* mutations were prevalent in CRC and polyp groups (52% and 32%, respectively). *KRAS* genes had 85% mutations confirmed and *BRAF* and *PIK3CA* confirmed in 100%. Mutations in more than one gene were also confirmed at 100%. Polyp group had 80% mutation confirmed to *KRAS* gene, but only 25% for *BRAF* gene.

In total of 110 fecal samples analyzed by KBP Array method, 28 had a mutation in one or more genes, 62 were wild-type and 20 were considered inconclusive (impossibility of classification results between mutated or WT). Mutations results showed 60.7% were in CRC and 35.7% in polyp group. *KRAS* mutations were prevalent in CRC and *BRAF* in the polyp group. 

Mutations were found in 30 (32.6%) tissues and 28 (31.1%) of stools. There was no statistical difference between mutation percentages or between genes mutated comparing tissues and stools.

*RanplexCRC Array versus KBP Array in Stool DNA*

RanplexCRC method had 48 stool samples analyses, 18 had mutations, 61.1% were in the CRC and in 20 samples, the results were inconclusive. Agreement, between the 2 methods studied, was assessed by Kappa index, where we observed that they had regular concordance rate between total results (44.5%). When comparing KBP with RanplexCRC, agreement percentage between the two methods for CRC increased to 64% (Table 2).

*Correlation between human DNA quantification and conclusive results prevalence by KBP and RanplexCRC Arrays*

Conclusive KBP Array fecal results had a higher HDNA mean (10.37ng/ul versus 0.037ng/ul inconclusive tests, p=0.001). RanplexCRC Array results also had a greater HDNA quantity (23.9 ng/ul) within conclusive tests compared to inconclusive tests (0.75ng/ul, p=0.049).

A ROC curve was performed to obtain a cutoff value of HDNA quantification. HDNA human with a greater predictive value was 0.386 ng/ul (95% CI: 0.7-0.9). Sensitivity and specificity analysis for cancer diagnosis was 66% and 82% respectively (Figure 1). For CRC diagnosis, tissue KBP Array had 55% sensitivity and 94% specificity, fecal KBP Array, 35% sensitivity and 96% specificity and RanplexCRC method had 78% sensitivity and 100% specificity (Table 3).

## Discussion

According to the Globocan 1.800.000 new cases of CRC and 881.000 deaths occurred (Bray et al., 2018). Although most studies shown decreased mortality from CRC after screening methods, studies should consider adherence to invasive tests, side effects and cost (Richter, 2008, Bevan and Rutter, 2018). 

In this study, feces were collected from individuals with prior request colonoscopy. Studies show that around 50% of individuals asked to collect stool for occult blood, perform collection (Grazzini et al., 2008, Larsen et al., 2018). In our study, approximately 70% of patients referred for colonoscopy were ready to bring a stool sample. 

Through molecular biology progress, new DNA stool tests have been developed with the purpose of early colorectal cancer or polyps detection. We found a relative high quality in total DNA among samples, indicating that most participants followed our guidelines. DNA amounts in feces between 70 and 300 ng/μl was considered optimal by extraction method chosen in this study.

CRC develop mainly from polyps, histologically classified as adenomas. Clinical features of individuals with polyps, had 75.8% adenoma polyps and 24.2% hyperplastic polyps. For many years this kind of damage was considered as non-neoplastic lesion. Bauer and Papaconstantinou (2008) called attention to hyperplastic polyps. According to these studies, such injuries account for 80 to 90% of serrated polyps. Microvesicular hyperplastic polyp subtype may present mutation in *BRAF* oncogene suggesting this type of injury as a precursor serrated adenoma that can be a CRC precursor (Sweetser et al., 2013).

In this study we used as the main material for analysis, DNA in feces. In cancer patients due to exfoliation of tumor cells average human DNA content (15.05±30.7 ng/ul) was approximately 33 times higher when compared to the control group. Similar results have already been published by our group (Teixeira et al., 2015). Among patients with polyps, no differences were observed when compared to the control group. These lesions appear to lose proliferative characteristics, and even cell adhesion apoptosis, but to a lesser extent when compared to tumor cells. 

This study intended to analyze known CRC mutations by microarray analysis and compare to DNA quantification in feces. KBP Array method was developed to evaluate tissue samples from patients with CRC. The array had never been assessed for stool or polyp samples and as such is not optimized for these sample types. Among tissue samples, mutations were observed in 53% of patients with CRC, 40% in polyp group and 7% in the control group. *KRAS* mutation was mainly found in 41% case group (Table 1). Mutations at codon 12 and 13 promote oncogenic *KRAS* gene potential and the most frequently described within the literature (Tsiatis et al., 2010). To confirm mutations, Sanger sequencing was performed. Array results were confirmed in 76% of cases. Genetic sequencing is considered a highly sensitive and specific methodology in genes study (Yamane et al., 2014). 

Using stool DNA in a method developed for tissue DNA was one of the biggest challenges of this study. As stated earlier, total DNA present in feces has a small percentage of HDNA, representing caution in handling, from extraction, storage, and manipulation. In most studies using stool DNA, researchers needed to “treat” DNA before analyzing it. This treatment mostly consists of amplification reaction for capturing human DNA in stool using specific sequences of nucleotides, the probes calls. In this work, a method not developed specifically for stool DNA samples, i.e. without possibility of capturing human DNA prior to genetic mutation analysis was used. Through KBP Array we observed 60.7% of mutations in cancer patients and 35.7% adenomatous or hyperplastic polyps. As observed in tissue, the most prevalent mutation was in *KRAS* in CRC (51.6%). In polyp group, *BRAF* gene mutation was most frequent (30.3%) (Table 2). Yamane et al., (2014) related two studies that demonstrated a high *KRAS* mutation frequency (45.2%) in serrated adenocarcinoma and suggest that a significant proportion of *KRAS* mutated CRC originates from serrated polyps and referenced high *BRAF* mutation frequency (V600E) among serrated carcinomas (82%), emphasizing that this mutation is a specific marker in the serrated pathway. 

We found 20 (18%) samples with inconclusive results, which can be explained by different amounts of human DNA present in feces of patients with and without cancer, 65% of patients belonged to the control group and only 15% from CRC. 

Genetic mutation analysis was also carried out with a method developed specifically for stool that identifies 28 mutations in four genes *(APC*, *KRAS*, *BRAF*, and *TP53*) involved in colorectal carcinogenesis, RanplexCRC Array. This method has as its main tool enrichment of specific regions of human DNA by PCR amplification of gene regions where mutations studied could be detected in other method steps. This assures researcher greater sensitivity for mutation analysis of DNA samples that are not purely human, as in stool DNA samples.

Unlike the KBPArray method, RanplexCRC stool DNA was developed as a CRC screen. Among 20 samples from patients with cancer, mutation presence was observed in a lower percentage (61%). Excluding inconclusive samples, mutation percentage was higher (78%). Assessing RanplexCRC staged method, we observed 36% mutations in *TP53*, *KRAS* or APC genes and 18% in *BRAF* gene. In polyp group, mutation rate was lower in RanplexCRC, the greatest number of inconclusive tests was in the control group (30%) (Table 3).

Comparing RanplexCRC and KBP Arrays in the feces, no differences were found between these two methods and there was a concordance of 64 % in CRC. These results suggest that KBP Array developed for CRC tissue analysis can also be used for stool DNA.

When performing sensitivity and specificity of methods excluding inconclusive results, we observed that KBP and RanplexCRC Array methods had a sensitivity of 35% and 78% for CRC. These results show that human DNA amount in stool is a key factor in colorectal cancer screening.

DNA quantity also can be related to mutations found, mainly in the KBP Array. With a cut-off of 0.4 ng/ul and we observed that from 28 mutations positive on stool DNA by KBP Array test, 25 had human DNA quantified and from this, 7 had less than 0.4 ng/ul and 18 more than 0.4 ng/ul. Sensitivity for this was 56%. 

In 2014, Imperiale et al., (2014) compared a panel of 21 mutations on stool DNA with hemoccult II and found 51.6% of sensitivity versus 12.9% by hemoccult II. Ahlquist et al., (2012) studied stool DNA methylation in 4 genes and mutation in *KRAS* gene found a sensitivity of 78% and specificity of 90% for CRC. According to Bosch et al., (2012) smaller number of cells would be required to detect DNA methylation in relation to mutation studies which increases diagnostic sensitivity. In polyps, authors found 48% of sensitivity for adenomas ≥ 1cm, unlike our findings, where sensitivity was between 63% and 71% for polyps, regardless of size, in the stool DNA study. Some stool DNA mutations considered as a false positive can also indicate a mistake of small tumor during colonoscopy.

The American Cancer Society (ACS) recommends as an alternative a screening study of stool DNA every 3 years (NCCN, 2018).

In conclusion, this study can contribute to CRC screening since Human DNA Quantification in fecal samples can have a low cost and simple method to allow cancer group identification. Microarray methods should be promised as a potential biomarker for colorectal cancer screening, given that KBP Array identified several mutations in precursor genes in stool DNA and it can be completed in under 3 hours via DNA input. However, there is a need to optimize these methods to improve accuracy and ensure applicability by clinical routine. 
